# A calibration of nucleic acid (PCR) by antibody (IgG) tests in Germany: the course of SARS-CoV-2 infections estimated

**DOI:** 10.3389/fepid.2025.1592629

**Published:** 2025-10-13

**Authors:** Michael Günther, Robert Rockenfeller, Harald Walach

**Affiliations:** ^1^ Computational Biophysics and Biorobotics, Institute for Modelling and Simulation of Biomechanical Systems, Universität Stuttgart, Stuttgart, Germany; ^2^ Friedrich–Schiller–Universität, Jena, Germany; ^3^ Mathematisches Institut, Universität Koblenz, Koblenz, Germany; ^4^ Change Health Science Institute, Basel, Switzerland; ^5^ Next Society Institute, Kazimieras Simonavicius University, Vilnius, Lithuania

**Keywords:** infectious disease, epidemiology, epidemic dynamics, COVID-19, serology

## Abstract

In Germany, a consortium of authority-accredited laboratories (ALM) covered approximately 90% of all severe acute respiratory syndrome coronavirus 2 (SARS-CoV-2) polymerase chain reaction (PCR) tests during the COVID-19 pandemic (March 2020 until January 2023), and they likewise conducted serological mass tests for IgG antibodies until May 2021. We analyzed the ALM-observed week-resolved time courses of test-positive fractions of PCR and IgG tests, by least-squares fitting a simple function of the former to the course of the latter. Specifically, we show that scaling and shifting the cumulative sum of previous PCR-positive fractions effectively reproduces the time course of the IgG-positive fraction. The value of 0.14 found for the fitted scaling parameter means that only 14% of those who were tested PCR-positively actually became infected with SARS-CoV-2. This parameter fit further implies that a quarter of the German population already carried IgG antibodies from natural infections in their blood at the turn of the year from 2020 to 2021. To check this fit using a second, independent analysis, we took from the literature the Germany-specific ratio of 1:10 for the ratio between one positive PCR test and the corresponding number of persons actually infected with SARS-CoV-2, and therewith estimated the time course of the latter within the German population. The courses of all three fractions, i.e., both the observed and the fit-estimated IgG-positives and the fit-estimated infected, matched each other well in the period from early December 2020 to May 2021. The extrapolated courses of both the fit-estimated fractions, i.e., those of the IgG-positives and the infected, align well to perfectly with the IgG-positive fraction (92%) reported by the Robert Koch Institute at the end of 2021.

## 1 Introduction

Public reporting of weekly polymerase chain reaction (PCR) test results provides a time-resolved signal of the detection of viral genetic material in a population, but does not directly quantify cumulative exposure. Here, we ask: To what extent can a summed PCR-positive signal be calibrated to reproduce the observed IgG seroprevalence trajectory (i.e., the IgG-positive signal)? We address this with two complementary, minimal models: (i) a least-squares fit that scales the cumulative weekly PCR-positive fraction to match positive IgG fractions and (ii) a literature-parametrized conversion from counts of positive PCR tests to an estimated number of infected in the population. These approaches are simple by design to maximize transparency and interpretability.

Detecting, through amplification, specific nucleic acid sequences of viral genes by conducting real-time quantitative reverse transcription PCR tests from a mucosal swab of a selected person proves the presence of viral genetic material at the epithelial–mucosal barrier, i.e., in the mucus coating the outside of the epithelial cells, within these cells, or in both layers. Active viral material entering the mucus or epithelial cells may be bound and possibly already neutralized by IgA antibodies, which are continuously produced by the body (1). IgA antibodies are by far the most prevalent antibody class, being the predominant Ig antibody at the epithelial–mucosal barrier and also constantly circulating in the blood, thus forming part of the humoral immune system. If upon *breaching* the epithelial–mucosal barrier—“breach” meaning the invasion of a person’s organism by active viral material—an increase in IgA concentration can also be detected as a response in the blood and it is common scientific terminology to say that “the person has become infected” (2). In most infection cases, particularly when symptoms occur, IgG antibodies will also become detectable in the blood (3) as a secondary immune response, which is somewhat delayed relative to the IgA activity at the epithelial–mucosal barrier. In fact, as a natural consequence of infection, the immune system initiates a multitude of responses, one of which is, besides the permanent presence of IgA antibodies both at the epithelial–mucosal barrier and in the blood, the production and secretion into the bloodstream (as part of the humoral immune system) of IgM antibodies in response to viral invasion of the interior of the body (i.e. the event of infection). The production and secretion of IgG antibodies (along with two others: IgD and IgE) then occurs with a delay of a few days compared to that of IgM antibodies. The presence of IgG antibodies in the blood is representative of the body’s *immunological memory* of infections.

Taking data on *severe acute respiratory syndrome coronavirus 2* (SARS-CoV-2; family *Coronaviridae*, genus *Betacoronavirus*) as a recent example, in more severe cases of illness, IgG antibodies become detectable approximately 2 weeks after infection, i.e., during the second week after symptom onset (3); in milder cases, it may take up to 4 weeks after infection (4, Supplementary Figure 1). According to another study (5), which performed IgG testing approximately half a year later, possibly with higher sensitivity than Rijkers et al. (4), even in cases with only mild or moderate symptoms, an immune response was detectable for at least 36 weeks after natural infection, and IgG antibodies remained detectable for up to a year in at least 90% of naturally infected SARS-CoV-2-IgG-positive individuals (6). Accordingly, over the course of a viral epidemic, the proportion of persons in a population who exhibit IgG antibodies in their blood indicates the cumulative share of the population who were previously infected or vaccinated. In other words, if a randomly selected *group* within the population is *tested for IgG antibodies*, then the *fraction* of that group *showing IgG antibodies*, i.e., those who are *IgG-positive*, *reflects* the total number of people infected up to 2 weeks (or even 7 days) prior (7).

After the emergence of SARS-CoV-2 in late 2019, PCR testing (8) for virus-specific genetic material in nasopharyngeal mucus became the global diagnostic gold standard. It is noteworthy that PCR tests merely detect the *presence of* fragments of viral genetic material, not necessarily an *active infection*. Nevertheless, it can be assumed that the detection of viral material at the epithelial–mucosal barrier correlates with a certain likelihood of infection. Therefore, the population-level IgG-positive fraction at any given testing time should be approximately *proportional to* the cumulative sum of fractions of individuals who tested PCR-positive until 2 weeks before the IgG test. This approximation only holds correctly if each PCR-positive person is tested positive just once during the analyzed period; in other words, PCR-positive *cases* should closely approximate *persons*. Indeed, strong quantitative evidence from a prior study by some of the present authors (9, Section 2.4) indicates that multiple testing was not widespread before late summer 2021, i.e., beyond the time frame primarily analyzed here (see Section 2).

Studying the relationship between PCR and IgG results is crucial, since PCR-positive counts were widely interpreted as proxies for actual infections and served as the basis for public health policy decisions. It is therefore important to highlight two known sources of false-positive PCR results. First, a study (10, ) found that the Charité’s PCR assay produced positive results on water controls at cycle threshold (CT) values between 36 and 38. Second, according to Bayes’ theorem, the rate of *false* positives increases when disease prevalence declines, owing to test specificity below 100%. In addition, individuals whose PCR tests require CT values above 30 are commonly not to be considered infectious (11, 12), whereas in practice, many tests were conducted with CT values up to 40 (13, 14), (15, Suppl.: CT ≤ 37), (16, CT ≤ 38), and even higher (8, CT=45).

In short, a PCR test provides a *snapshot* of an individual’s *current exposure* to viral genetic material at the outermost layers of the body. In epidemiological terms, the PCR-positive fraction can be interpreted as a (proportional but not equal) proxy for the normalized incidence of viral infections, specifically SARS-CoV-2 in this case. Contrary to the definition of “incidence” in the German Infection Protection Act § 28a(3) (“Infektionsschutzgesetz”), this fraction does not depend on the absolute number of tests conducted (assuming *invariant* testing conditions, though selection effects may occur, e.g., by targeting), and is therefore a more robust indicator of infection frequency. In other words, when normalized to the number of tests, the incidence identifies the denominator as the varying number of people tested, rather than a fixed group size (e.g., a district population). Although this adjustment does not address pre-selection biases (e.g., symptom-based testing), such biases affect the representativeness of any test-positive fraction; this issue is discussed below. In contrast, virus-specific IgG tests assess whether a specific virus has previously (within a memory window spanning months to years) infected the individual; vaccination, too, typically induces both IgM and IgG production. Accordingly, the IgG-positive fraction reflects the share of the population that has previously been infected or vaccinated, and thus serves as evidence of collective immune response. Mathematically, the IgG-positive fraction at a given time should be proportional to the accumulated PCR-positive incidence, at least during the first year after the virus’ emergence. This ignores the small error margin due to IgG test sensitivity limitations (17), which range from 80% to 81% (18), 83% to 86% (7), 91% (19), 97.5% (6), and up to 100% (20), and also accounts for “negative” seroconversion events (16, 21, 22), with *both* factors causing underestimation of true infection rates.

Consequently, as the main objective of this study, we examine to what extent the observed PCR-positive normalized incidence—serving as a weekly snapshot of viral detection in the population—fits the observed (and potentially population-representative) IgG-positive normalized fraction, which reflects the immune system’s memory of past infections. Both datasets are based on measurements reported by the same authority-accredited laboratories. To investigate this relationship, we apply a least-squares fit using two parameters to model the connection between the PCR- and IgG-positive fractions through a simple phenomenological function (model 1), effectively yielding an epidemiological calibration. In a second, independent step, we use the time series of observed raw PCR-positive test counts—together with three parameter values taken from the literature—to estimate the trajectory of the actually infected fraction within the entire German population (model 2). This second approach assumes that the IgG-positive fraction is a practical proxy for prior infections, and thereby serves to validate the results of model 1.

## 2 Materials, methods, and results

We examined the week-resolved relationship between the cumulative sum of the fraction of positive PCR test counts and the corresponding fraction of positive IgG test counts over the specific period from mid-March 2020 until the end of 2021 in Germany. The data were obtained from a webpage (23), where a medical laboratory consortium (*Akkreditierte Labore in der Medizin e.V.*, ALM, Berlin, Germany) reported weekly PCR and IgG test results from German test laboratories, including both absolute numbers and proportions of positive outcomes. The ALM dataset consists of weekly aggregated counts, that is, the demographic information reported was not stratified by, for example, age or sex. It is noteworthy that the online data source (23) is no longer available. Only parts of the IgG dataset are still accessible via (24), primarily in the form of printed tables occasionally included in press briefings, and only up to the final calendar week (#53) of 2020. However, we previously extracted and saved the full dataset as displayed in interactive online graphic panels (23); it is provided in terms of two separate files (PCR and IgG data, respectively) as Supplementary Material (25).

**Figure 1:**
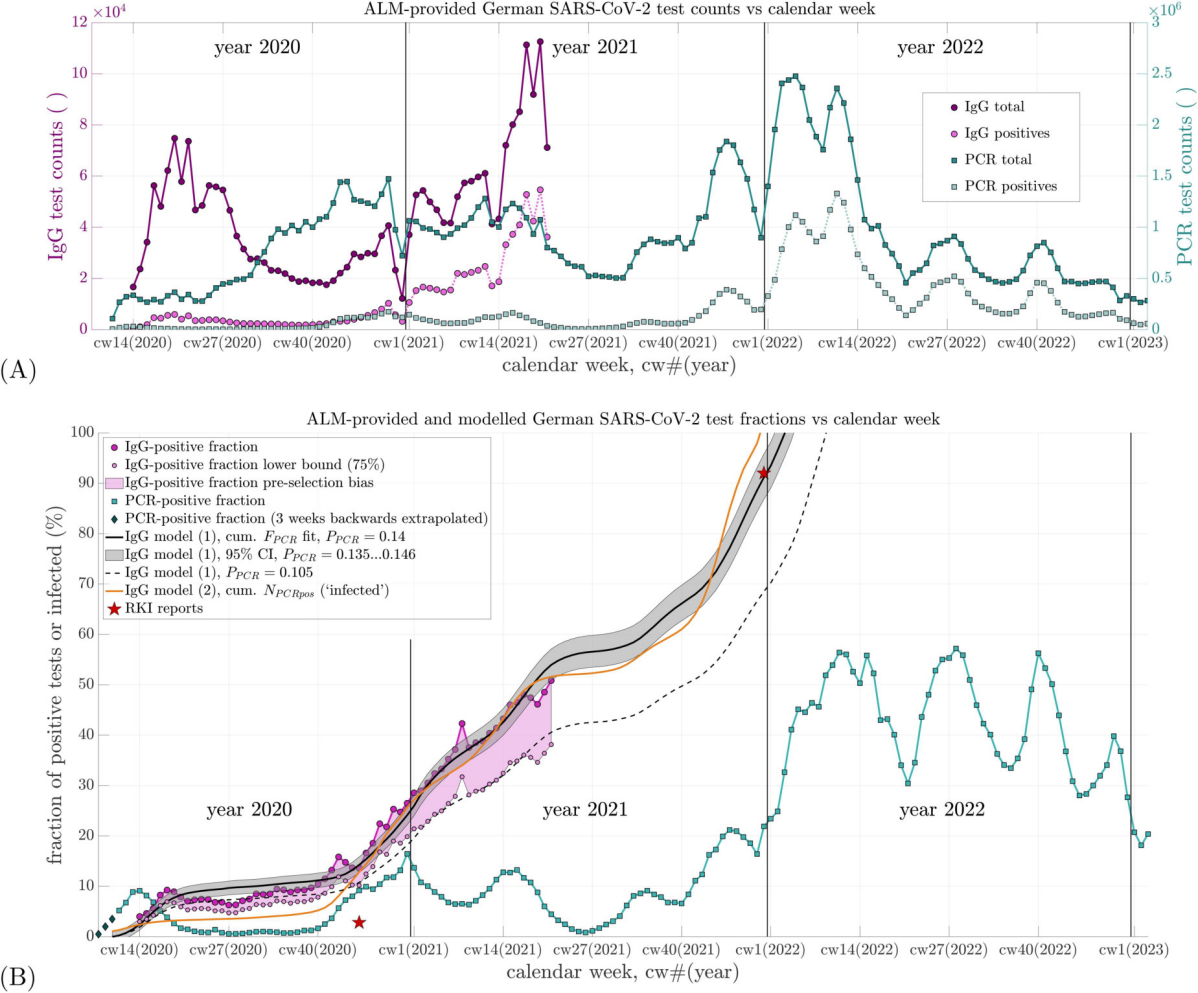
**(A)** Weekly counts of total SARS-CoV-2 PCR and IgG antibody tests conducted by ALM Laboratories in Germany from 2020 to 2022, along with their respective positive share. The left vertical axis applies to IgG test counts; the right vertical axis applies to PCR test counts. The time axis (abscissa) begins at calendar week 08 of 2020 [cw08(2020)], which also applies to subfigure **(B)**. **(B)** Percent positive rates (fractions of positive tests relative to total tests) calculated from the data in **(A)** are shown for both PCR and IgG tests, represented by the topmost and fourth entries in the legend, respectively. The second and third entries depict a lower-bound estimate of the IgG-positive fraction, visualizing potential data uncertainty due to pre-selection effects (e.g., symptom-based testing). A maximum potential bias factor of 75% is estimated based on Tancredi et al. (33, Figure 2). The fifth item shows three linearly back-extrapolated data points extending the PCR-positive fraction to cw08(2020). The sixth item (black line) represents the best-fit IgG-positive fraction based on Equation 1 using $P_{\mathrm{PCR}}=0.14$. The seventh item (grey shaded area) corresponds to the 95% confidence interval around this optimal fit. The eighth item (black dashed line) shows the fit to the lower-bound IgG-positive estimate using $P_{\mathrm{PCR}}=0.105$. The ninth item (orange line) displays the estimated fraction of SARS-CoV-2-infected individuals according to Equation 2, with three of four parameters derived from the literature and one (non-sensitive) initial value reasonably assumed as representative of early infection levels. The tenth item (red stars) indicates two IgG-positive (or infection) prevalence estimates reported by the RKI (31, 32, 34, 35).

The ALM consortium continuously conducted approximately 90% of all PCR tests in Germany (24). Their weekly total test counts are displayed in Figure 1A (dark turquoise squares) against calendar weeks (cws), where cwX(Y) denotes calendar week X in year Y, e.g., cw10(2020), referring to the week ending 8 March 2020. The corresponding weekly number of PCR tests yielding positive results is shown as light turquoise squares.The total weekly number of IgG tests and those testing positive are plotted as dark and light magenta circles, respectively, in Figure 1A. The weekly positivity ratios observed by ALM, i.e., the fractions of positive PCR and IgG tests relative to total tests conducted, are shown as percentages in Figure 1B: turquoise squares denote the PCR-positive fraction, and magenta circles the IgG-positive fraction.

In the following, we introduce two complementary, minimal models, with simple, and thus transparent, equations: one (model 1) that scales the cumulative weekly PCR-positive fraction to match positive IgG fractions and one (model 2) that converts, with parameters known for Germany from the literature, counts of positive PCR tests to an estimated number of infected persons.

Besides showing the two positive test fractions in Figure 1B, the trajectory of the cumulative sum of prior PCR-positive fractions is also plotted, scaled by a multiplier to best match (fit) the ALM-observed IgG-positive fraction; this summing-and-scaling is our first of the two simple model estimates: model 1. To estimate the IgG-positive fraction at a given cw by model 1 (Equation 1), the PCR-positive fractions from earlier weeks are summed up to 2 weeks prior to that cw. This sum is then scaled by a factor $P_{\mathrm{PCR}}$, and an offset $O_{\mathrm{IgG},0}$ is added, representing the IgG-positive fraction 2 weeks after the start of summation. The modeled IgG-positive fraction in calendar week cw is thus given by (model 1)

(1) $$F_{\mathrm{IgG},\mathrm{cw}}=O_{\mathrm{IgG},0}+P_{\mathrm{PCR}}\cdot \sum_{i=8}^{\mathrm{cw}-2}F_{\mathrm{PCR},i}.$$

Here, $F_{\mathrm{PCR},i}=N_{\mathrm{PCRpos},i}/N_{\mathrm{PCR},i}$ is the *observed* PCR-positive fraction in calendar week i until cw−2, with $N_{\mathrm{PCR},i}$ and $N_{\mathrm{PCRpos},i}$ representing the weekly counts of PCR tests conducted and those yielding positive results, respectively. The summation in Equation 1 begins at i=8 (see end of this paragraph), hence requiring cw−2≥8, i.e. cw≥10. Equation 1 assumes that IgG antibodies become detectable 2 weeks after infection, and PCR-positive data are available starting from cw8(2020) (i=8).

In Equation 1, $O_{\mathrm{IgG},0}$ denotes the *estimated* IgG-positive fraction at cw10(2020), i.e., $F_{\mathrm{IgG},\mathrm{cw}=10}$. The symbol $F_{\mathrm{IgG},\mathrm{cw}}$ represents the *estimated* IgG-positive fraction for calendar week cw. The IgG-positive fractions $F_{\mathrm{IgG},i}=N_{\mathrm{IgGpos},i}/N_{\mathrm{IgG},i}$ *observed* for each cw i are derived from the weekly IgG test counts $N_{\mathrm{IgG},i}$ and $N_{\mathrm{IgGpos},i}$. Using the “lsqnonlin” function in MATLAB (Version R2024b, The MathWorks, Natick, MA, USA), the estimated sequence $F_{\mathrm{IgG},\mathrm{cw}}$ defined in Equation 1 was least-squares fitted to the observed sequence $F_{\mathrm{IgG},i}$, optimizing the parameters $O_{\mathrm{IgG},0}$ and $P_{\mathrm{PCR}}$.

The ALM reported their first PCR data point in cw11(2020), the week ending 15 March 2020. To capture the full scope of the initial SARS-CoV-2 wave, we linearly back-extrapolated the PCR-positive fraction $F_{\mathrm{PCR},i}$ for 3 weeks: cw8(2020) [end: 23 February]: 0.005; cw9(2020) [end: 1 March]: 0.02; cw10(2020) [end: 8 March]: 0.035 [see dark gray diamonds in Figure 1B, labeled “PCR-positive fraction (3 weeks backwards extrapolated)”], which implies that the PCR-positive fraction was very low or negligible until cw7(2020).

Our main parameter finding from fitting Equation 1 to the observed IgG data $F_{\mathrm{IgG},i}$ was $P_{\mathrm{PCR}}=1/7.15\approx 0.14$ (confidence interval, CI: 0.135–0.146). This suggests that, on average, only approximately 14% of those who tested PCR-positive were actually infected. The fitted offset $O_{\mathrm{IgG},0}$ was −0.001, i.e., −0.1%≈0 (CI: −1.1%to0.9%). With $P_{\mathrm{PCR}}\approx 0.14$, the model estimate $F_{\mathrm{IgG},\mathrm{cw}}$ [Figure 1B, black line: “IgG model (1), cum. $F_{\mathrm{PCR}}$ fit, $P_{\mathrm{PCR}}=0.14$”] fits the ALM-observed IgG data points $F_{\mathrm{IgG},i}$ (magenta circles: “IgG-positive fraction”) well. The mean residual per sample (i.e., root mean square error over 61 samples) was 2.2%.

This model 1 estimate $F_{\mathrm{IgG},\mathrm{cw}}$ further allows extrapolation beyond the ALM IgG data, which end at cw21(2020), extending until early 2022 when the modeled IgG-positive fraction reaches 1. The result $P_{\mathrm{PCR}}\approx 0.14$ implies that only one in approximately seven PCR-positive individuals was actually infected. This interpretation is based on a key *assumption*, made due to a lack of *a priori* knowledge regarding selection criteria for IgG testing: we *assume*, for the moment, that those tested for IgG were drawn from among those previously PCR-tested. However, as discussed in Section 3.1, this assumption is almost certainly incorrect. In reality, the ALM-reported IgG-positive fraction is close to population-representative, which lends further transparency to the analysis in Equation 1. The pre-selection bias inherent to PCR testing thus remains unquantified, but is effectively encapsulated by the proportionality factor $P_{\mathrm{PCR}}$ in a phenomenological sense.

As a result, whether one assumes that the IgG-tested individuals were drawn from the PCR-tested population or that they were broadly population-representative, the outcome remains the same: only approximately 14% of all PCR-positive individuals were actually infected with SARS-CoV-2, according to ALM data. This holds regardless of the intransparency of the pre-selection criteria for those PCR-tested (e.g., preceding an antigen test, contact-traced, or with clinical symptoms) and of those IgG-tested very likely being general practitioners’ patients who enquired about their immune status, yet, evidently being close to population-representative (see Section 3.1).

Second, we estimated using model 2 (Equation 2) the time course of the fraction $F_{\mathrm{infec},\mathrm{cw}}$ of SARS-CoV-2-infected individuals within the entire German population ($N_{\mathrm{pop}}=83.5\times 10^{6}$ inhabitants). To do this, we transformed the statement “For each positive PCR test, there are approximately 10 actual infections,” which subsumes empirical findings in Germany (26, $U_{\mathrm{infec}}\approx 10$) and Switzerland (27, 28, SEROCoV-POP: $U_{\mathrm{infec}}\approx 11$) into another simple equation (model 2)

(2) $$F_{\mathrm{infec},\mathrm{cw}}=O_{\mathrm{infec},0}+\frac{U_{\mathrm{infec}}}{R_{ALM}}\cdot \frac{\sum_{i=0}^{\mathrm{cw}}N_{\mathrm{PCRpos},i}}{N_{\mathrm{pop}}}.$$

In Equation 2, the parameters are as follows: $O_{\mathrm{infec},0}=0.01$ is the assumed baseline fraction of infected individuals at cw10(2020), $U_{\mathrm{infec}}=10$ is the empirical estimate of the number of infections per PCR-positive test, and $R_{\mathrm{ALM}}=0.9$ reflects ALM’s share (90%) of all PCR testing in Germany (24). This value of $U_{\mathrm{infec}}=10$ is also consistent with the approximate ratio between the infection fatality rate (IFR) and the case fatality rate (CFR) for Germany: CFR/IFR≈10, with CFR≈0.025 (9, $r_{\text{PF}}$ in Tables 3, 4), and IFR≈0.0021–0.0025 (29, “Germany” in Table 4).

Since 1942 (30), the detection of virus-specific antibodies has been regarded as the methodological gold standard for confirming infection. Accordingly, Equation 2 (model 2) represents an alternative means of estimating the antibody-positive fraction; compared with Equation 1 (model 1), it only requires as an input the weekly counts of PCR tests, rather than also the positive PCR test counts for assigning the positive fraction. Therefore, Equation 2, with its parameter values taken as either unambiguously countable or phenomenologically descriptive ones from the literature (all different from the input to Equation 1), encompasses a perspective, a methodical approach, and possibly a potential meaning distinctly different from Equation 1. It can thus be considered an independent approach to validate model 1 (via the output from Equation 1) by model 2 (via the output from Equation 2), although, when viewing it from a purely mathematical perspective, Equations 1 and 2 partly share the same input data (the sequence of weekly samples of PCR test counts). The time course of $F_{\mathrm{infec},\mathrm{cw}}$ as estimated by Equation 2 is shown as an orange line in Figure 1B and labeled “IgG model (2), cum. $N_{\mathrm{PCRpos}}$ (‘infected’).” From November 2020 onward, the curve lies approximately 2%–6% below the observed IgG-positive fraction and the fitted estimate $F_{\mathrm{IgG},\mathrm{cw}}$ from model 1. From cw46(2021) onward, however, it surpasses $F_{\mathrm{IgG},\mathrm{cw}}$ as extrapolated toward the end of 2021.

At the turn of the year 2021/2022, the extrapolated value of $F_{\mathrm{IgG},\mathrm{cw}}$ reaches approximately 92%, matching the value reported by the Robert Koch Institute (RKI) (31, 32). Meanwhile, the modeled infected fraction $F_{\mathrm{infec},\mathrm{cw}}$ reaches its theoretical maximum of 100%. The steep increase in $F_{\mathrm{infec},\mathrm{cw}}$ at the end of 2021 results numerically from the peak in PCR-positive test counts around cw47(2021), as shown in Figure 1A. The apparent attainment of 100% suggests that, by late 2021, multiple PCR tests were being conducted per individual, deviating from the assumption of one test per person. Evidence of this phenomenon is discussed in the study by Rockenfeller et al. (9, Section 3.3; Tables 5, 6). Thus, the steep rise and eventual overtaking of $F_{\mathrm{IgG},\mathrm{cw}}$ by $F_{\mathrm{infec},\mathrm{cw}}$ cannot be regarded as a reliable finding. Nevertheless, considering $F_{\mathrm{IgG},\mathrm{cw}}=0.92$ in cw52(2021) and the trajectory of $F_{\mathrm{infec},\mathrm{cw}}$ reaching approximately 65% by cw43(2021), we conservatively estimate that by the end of 2021, at least 85% of the German population had been infected at least once with SARS-CoV-2—a figure in close agreement with the 92% reported by the RKI.

A particularly noteworthy implication of the fitted IgG data is the rate at which the IgG-positive fraction increased during the first half of 2021, coinciding with the anti-SARS-CoV-2 injection campaign, with the IgG-positive fraction increasing at an average rate of 1.1% per week. This rate averaged 1.1% per week, corresponding to the slope of a least-squares linear fit through the final 12 data points of $F_{\mathrm{IgG},i}$ [i.e., magenta circles up to cw21(2021) in Figure 1B]. By contrast, during the final weeks of 2020—prior to the start of the anti-SARS-CoV-2 injection campaign—the slope of the observed IgG-positive curve [from cw45 to cw52(2020)] was steeper, averaging approximately 1.8% per week, and driven entirely by natural infections.

## 3 Discussion

The main findings of our analysis are listed below:

• Fitting the scaled cumulative PCR-positive fraction (model 1) of persons tested for SARS-CoV-2 to the ALM-observed SARS-CoV-2 seroprevalence trajectory (i.e., the time course of the IgG-positive fraction) yields $P_{\mathrm{PCR}}\approx 0.14$ (95% CI: 0.135–0.146) as a calibration factor for Germany. This implies that roughly only one in seven German individuals with a PCR-positive test later had detectable IgG antibodies, that is, was actually infected with SARS-CoV-2.
• A separate, count-based model 2 using $U_{\mathrm{infec}}=10$ and $R_{ALM}=0.9$ produces a SARS-CoV-2-infected fraction trajectory that is broadly consistent with the $P_{\mathrm{PCR}}$-scaled SARS-CoV-2-IgG-positive curve (i.e., with model 1), and both model estimates approach the RKI-reported aggregate IgG fraction of approximately 92% by the end of 2021.
• The key limitations are the aggregated nature of ALM data (no age or sex strata available) and the possible (but moderate in its effect) pre-selection bias in who was IgG-tested.

### 3.1 Sensitivity of inferences on the population’s course of infections: considering pre-selection bias in testing persons

Our data analysis and its interpretation in this study have taken the ALM-reported PCR-positive fractions as given; these data inherently reflect all pre-selection factors of the German testing strategy, such as preferentially testing individuals with symptoms or known contact with confirmed cases. To the best of our knowledge, no data have been published for Germany that would allow quantification of this (certainly time-dependent) pre-selection bias in the observed PCR data. By “bias,” we refer to the ratio between the observed test-positive fraction and the theoretical value that would be obtained under truly population-representative, random sampling. A relevant comparison can be drawn from Belgian schools, where targeted testing introduced a net bias factor of approximately 3, as shown by comparing PCR positivity (“PR”) among all tested individuals with that in randomly “screened” subgroups (36, Figure 4).

Building on this, we begin by addressing an obvious objection to directly estimating the proportion of SARS-CoV-2-infected individuals in the population by linearly relating lab-reported PCR- and IgG-positive fractions from weekly sub-populations of tested individuals in Germany. This objection stems from the possibility that ALM’s IgG testing itself may also be affected by pre-selection. If so, a person who is genuinely population-representative may have had a *lower* probability of testing IgG-positive than someone tested within the ALM’s sub-clientele. Conversely, the true IgG-positive fraction in the general population may have been somewhat *lower* than what the ALM observed. As a consequence, the proportionality factor $P_{\mathrm{PCR}}$ in Equation 1 would also be expected to be *lower* than the fitted value of 0.14. In Figure 1B, we visualized a potential lower bound on the IgG-positive fraction by applying a maximum bias correction factor of 75% to the observed ALM data. This factor was estimated from the literature (33, Figure 2) by roughly averaging the regional deviation factors.

In summary, any pre-selection bias in the ALM-observed IgG-positive fraction would have being tending to *overestimate* the proportion of truly infected individuals in the German population. Consequently, if the proportion of infected had in fact been *lower* than that observed by the ALM, then the $P_{\mathrm{PCR}}$ value estimated from the fit by Equation 1 would likewise have to be even *lower* than 0.14. Accordingly, a more conservative interpretation of our results suggests that as few as one in eight or even in nine PCR-positive individuals, i.e., approximately 11% ($P_{\mathrm{PCR}}<0.105$), may have actually been infected, rather than one in seven (14%, $P_{\mathrm{PCR}}=0.14$).

Further, as an independent validation of Equation 1, we refer to the additional analysis presented in the second half of Section 2, which introduces Equation 2. This equation provides a complementary estimate of the (population-representative) IgG-positive fraction using literature-derived parameters. Notably, both the PCR-based model estimates of the IgG-positive fraction yield values that closely match the RKI-reported 92% IgG seroprevalence value by the end of 2021 (31, 32). First, this agreement is found in the extrapolated IgG-positive curve based on our fitted proportionality parameter $P_{\mathrm{PCR}}$ (via Equation 1). Second, it is reflected in the time course of the infected population as estimated using the infection multiplier $U_{\mathrm{infec}}$ derived from the literature (via Equation 2). Moreover, the value $U_{\mathrm{infec}}=10$ is independently consistent with the ratio between the CFR (9) and IFR (29) values found by analyses of German epidemiological data.

Altogether, the cross-check between Equation 1 and the literature-based formulation in Equation 2 provides strong support for the conclusion that the ALM-reported IgG-positive fraction is indeed closely representative of the true infection dynamics in the German population.

### 3.2 Conditions of testing, PCR and IgG thresholds, and PCR test specificity

In our view, the result of comparing the $P_{\mathrm{PCR}}$-scaled cumulative sum of observed PCR-positive fractions (Equation 1) with the observed IgG-positive fraction is striking. Given the sheer simplicity of the summation model—which implicitly abstracts both physiological details, such as virus presence at epithelial–mucosal barriers and serologically detectable antibody responses of the humoral immune system, and technological details, such as testing procedures and laboratory parameters—the agreement between the modeled IgG-positive time course and the directly observed one (Figure 1B) can be regarded as surprisingly close.

The systematic underestimation and subsequent overestimation of the ALM-observed IgG-positive fraction by the $P_{\mathrm{PCR}}$-scaled cumulative PCR signal during spring and summer 2020 may reflect changes in governmental testing policy. From early May 2020 onward, testing was expanded to include asymptomatic individuals (so-called “mass testing”). Later in the summer, antibody levels may have begun to decline below detection thresholds, contributing to observed discrepancies. Another possible explanation for the overestimation of IgG-positivity by the model (particularly from May to September 2020) is that the sensitivity of early SARS-CoV-2 antibody test kits (e.g., ELISA) was lower than that of kits used later in the year. In addition, the temporary overestimation peak observed in late April 2020 may be attributable to strong pre-selection biases, whereby individuals tested for IgG were more likely to have had a prior positive PCR test. This effect was likely reduced in the following months (May and June) as PCR test volumes increased at a time of very low prevalence (see Figure 1A).

Naturally, a PCR-positive test alone can by no means confirm infection at the individual level. The fitted proportionality factor $P_{\mathrm{PCR}}=1/7.15\approx 0.14$ indicates that only a minority of PCR-positive individuals were actually infected. This factor, $P_{\mathrm{PCR}}$, is in itself the net result of multiple multiplicative influences—most notably: (i) time-varying and non-standardized pre-selection criteria for testing (e.g., symptomatic screening), (ii) non-uniform CT thresholds applied by laboratories in PCR analysis, and (iii) varying detection methods and thresholds in IgG testing (e.g., reagent concentrations, optical density cut-offs). All of these effects—beyond pre-selection—are generally subsumed under the two core parameters of diagnostic testing: sensitivity and specificity [see summary in Watson et al. (37)]. In essence, $P_{\mathrm{PCR}}$ reflects the net probability that a person will become serologically IgG-positive (i.e., has been infected) if SARS-CoV-2 genetic material is detectable by PCR at the epithelial–mucosal barrier. This probability is estimated to be approximately 14% (CI: 13.5%–14.6%), with a conservative lower bound of 10.5% if ALM IgG data are assumed to overestimate the population-representative level.

The test performance parameter most relevant to our findings is the specificity of PCR mass testing in Germany during 2020 and 2021. Regardless of PCR sensitivity (which we may, for argument’s sake, assume to be 100%), the combination of observed parameters allows for an estimation of specificity. The mean weekly PCR-positive fraction in Germany between cw11(2020) and cw21(2021), i.e., the ALM IgG testing period, was approximately 7%. Meanwhile, the fitted $P_{\mathrm{PCR}}=1/7.15$ implies that only approximately 1% of those tested per week were actually infected. Assuming 1% of tested individuals were true positives, a specificity of 94% explains the remaining 6% of PCR-positive results as false positives among the 99% who were not infected. This estimate is in excellent agreement with direct assessments of PCR specificity in the literature (38, ).

In summary, our finding that $P_{\mathrm{PCR}}=1/7.15$ is entirely consistent with both the observed low PCR-positive rates and an overall PCR specificity of 94% in Germany. This interpretation provides a coherent picture of the relationship between infection status and test positivity during mass testing.

### 3.3 IgG-positive fractions: comparison of laboratory observations, estimated population infections, RKI-reported data, and the literature

Utilizing the two largely independent model approaches described above, we estimated the fraction of SARS-CoV-2-infected individuals at the onset of Germany’s anti-SARS-CoV-2 injection campaign on 27 December 2020 (see Figure 1B) to account for approximately 24%. This is striking, because the RKI in contrast reported that no more than 2.8% (34)—and in earlier statements not even more than 2% (35)—of the population had been IgG-positive “until November 2020.” The specific cut-off date for this percentage was not provided. If we assume mid-November, i.e., cw46(2020), the ALM-observed IgG-positive fraction stood at 15%, a value fully corroborated by our modeled estimate via the $P_{\mathrm{PCR}}$-scaled cumulative PCR-positive signal (Equation 1). Moreover, our separate estimate of the population-wide cumulative infection rate based on Equation 2 was 11% at the same time. This discrepancy between, on the one hand, both the ALM-observed and modeled IgG-positive fractions and, on the other hand, the substantially lower RKI-reported values is remarkable—especially considering that the ALM data were not only available to but in fact commissioned by the RKI. One likely explanation is that the serological method used in the RKI-SOEP study—self-collected dried blood spot samples—was insufficiently sensitive (34).

As for the difference between the ALM-observed IgG-positive fraction and our generally lower estimate of the SARS-CoV-2-infected proportion (Equation 2), specific information regarding the IgG testing methods used by ALM or on how test subjects were selected were not available—apart from the general note that all tests were requested by physicians (24). This implies that IgG testing likely took place during patient consultations, regardless of the medical reason. Despite this lack of detail, the ALM dataset is substantial, comprising 12,000–100,000 IgG tests per week (see Figure 1A) across Germany. It is unclear why ALM ceased reporting IgG data after cw21(2021), especially as the IgG-positive fraction had just reached 50%. Notably, by the end of 2021, the RKI reported a national IgG-positive rate of approximately 92% (31, 32). This value aligns remarkably well with both our extrapolated ALM-based IgG estimates (via Equation 1) and our modeled estimate of the total infected fraction based on Equation 2. Moreover, our estimated time courses for both IgG-positive and infected fractions in Germany (see Figure 1B) lie well within the range observed for regional populations in Switzerland, as reported by the *Corona Immunitas* initiative (33, Figure 2), which employed a systematic sampling design aimed at representing the entire Swiss population.

Additional seroprevalence studies further validate our estimates. According to Piler et al. (39, Figure 1), the IgG-positive fraction in the Czech population was approximately 8% in early October 2020, rising to 16% by the end of that month, 35% in November, approximately 42% in December and January 2021, and approximately 56% by the end of March 2021. These trends closely match Germany’s three-wave PCR-positive curve, albeit occurring approximately 4 weeks earlier in the Czech Republic. In Pakistan, a study reported average IgG-positive rates of 23% in July–August 2020, 28% in October–December 2020, 48% in February–April 2021, and 78% in September–November 2021 (40, Figure 2). Similarly, an observational study of Belgian pupils and teachers reported IgG-positive fractions up to 62% in December 2021 (41).

Finally, the World Health Organization (WHO), the German Federal Ministry of Health (BMG), and the RKI are among the sponsors of the “Serotracker” project (42), which aims to provide a global overview of SARS-CoV-2 antibody studies. As of June 2025, several of the studies cited above (33, 34, 39) are indexed in the platform, although others—such as Iqbal et al. (40)—are not. While Serotracker is a valuable resource, its use requires caution: the platform often displays only one specific seroprevalence value in its pop-up interface, whereas the underlying publications may contain more extensive datasets.

## 4 Summary and conclusion

The principal finding from our analysis of ALM data on both nucleic acid amplification (PCR from mucosal swabs) and IgG antibody (serological) testing for SARS-CoV-2 in Germany between mid-March 2020 and summer 2021 is this: only 14%—and possibly even fewer, down to 10%—of individuals identified as SARS-CoV-2-positive via PCR testing were actually infected, as evidenced by detectable IgG antibodies.

Our conclusion is twofold. First, the IgG testing conducted by ALM laboratories was commissioned by the RKI, itself subordinate to the BMG. Nonetheless, data acquisition evidently ceased after cw21(2021) or, at the very least, public reporting of the data on the ALM website (23) stopped. The IgG results observed and published by ALM have not been acknowledged or communicated by the RKI to date, despite the fact that transparency in reporting such data should be mandatory, both scientifically and in terms of public accountability. Second, the proportion of the German population with a detectable immune response to SARS-CoV-2 was already substantial by the end of 2020. Approximately one-quarter of the population carried IgG antibodies at that point, following a trajectory determined almost exclusively by natural infections. By the end of 2021, practically the whole German population could be considered IgG positive.

Evidently, from March 2020 onward, a national German serological antibody cohort study was conducted—initiated and overseen by the RKI and BMG—though it was never publicly communicated as such, nor has it been adequately analyzed to this day. In consequence, German authorities had timely and reliable access to data tracking the course of IgG seropositivity—data that were, in fact, close to being population-representative. These data could have served as an objective metric for monitoring the proclaimed “epidemic situation of national significance” (“Epidemische Lage Nationaler Tragweite”).

Instead, this evidence-based and representative serological signal was disregarded in favor of relying on the weekly *absolute* number of positive PCR tests—the so-called “7-day incidence” (“Sieben-Tage-Inzidenz”). Unequivocally, this definition of incidence yields a scientifically meaningless figure in the context of infection dynamics, as it depends entirely on the arbitrary (or imposed) number of PCR tests performed. It is therefore not an objective indicator of epidemiological reality, but an administratively imposed figure—more reflective of political will than scientific rigor. Yet, incomprehensibly, this 7-day incidence metric was even incorporated into the German Infection Protection Act (“Infektionsschutzgesetz”) as the quantitative foundation for imposing highly restrictive public health measures. The methodological shortcomings and institutional processes that enabled its elevation to policy status demand critical re-evaluation—not only to prevent similar errors in the future, but to restore trust in evidence-based public health governance.

## Data Availability

The original contributions presented in the study are included in the article/**Supplementary Material**, further inquiries can be directed to the corresponding author.
