# Supplementary material for: A calibration of nucleic acid (PCR) by antibody (IgG) tests in Germany: the course of SARS-CoV-2 infections estimated
Source: Front Epidemiol. 2025 Oct 13;5:1592629. doi: 10.3389/fepid.2025.1592629 (PMC12554765; doi:10.3389/fepid.2025.1592629)
Supplement: Supplementary file 1 [file Datasheet1.zip › Supplement/ALM-Daten_IgG_und_PCR_Corona-Diagnostik_Insights.pdf]

CoronaDiagnostikInsights.

Daten, Zahlen und Fakten zur SARS-CoV-2-Diagnostik der Labore in Deutschland

Hintergrund

Der Verband der Akkreditierten Labore in der Medizin, ALM e.V., führt seit Anfang März in Abstimmung mit den Behörden auf Bundesebene eine strukturierte und standardisierte Datenerhebung durch. An der Datenerhebung beteiligen sich bundesweit 179 Labore aus dem gesamten Bundesgebiet, die ca. 90 Prozent des aktuellen Corona-Testgeschehens aus allen Bereichen repräsentieren.

Weitere aktuelle Informationen finden Sie auch hier:

Bundesministerium für Gesundheit (BMG)

Zusammen gegen Corona (BGM / BZgA / RKI)

Robert-Koch-Institut (RKI)

Kassenärztliche Bundesvereinigung (KBV)

GKV-Spitzenverband

Die Daten dienen Politik, Krisenstäben und dem Robert-Koch-Institut (RKI) als eine wesentliche Grundlage zur Beurteilung des Testgeschehens. Die Ergebnisse werden an das RKI übermittelt und dort mit zusätzlich vorhandenen Daten zusammengeführt. Die Gesamtübersicht wird im RKI-Lagebericht veröffentlicht.

Website des ALM e.V.

Wir weisen ausdrücklich darauf hin, dass die hier ermittelten Daten mit weiteren Daten am RKI zusammengeführt werden. Die daraus entstehenden Daten stellen das Gesamtbild über das Testgeschehen in Deutschland dar. Eine anderweitige Nutzung der Daten darf nur mit Hinweis auf die Erhebung des ALM e.V. als Quelle erfolgen.

Verlauf der SARS-CoV-2-PCR Testungen

SARS-CoV-2-PCR Tests: Kapazitäten, Anzahl der Testungen, positive Ergebnisse

interaktives Diagramm

Letzte 10 Wochen | Verlauf 2021 | Verlauf 2020

Abbildung 1: Die Testkapazität der teilnehmenden Labore liegt aktuell bei ca. 2.010.000 Tests pro Woche. Die Kapazität der Facharztlabore reicht für den medizinischen Bedarf an Tests sicher aus.

Stand 26.10.2021

Verlauf der IgG-Antikörpertestungen

interaktives Diagramm

Letzte 10 Wochen Verlauf 2021 Verlauf 2020

Abbildung 2: Immunglobulin-G (IgG)-Antikörpertests werden zum Nachweis einer vorausgegangenen SARS-CoV-2-Infektion eingesetzt. Die Serokonversion (Antikörpernachweis in Verlaufsuntersuchungen) zeigt eine Infektion an. Aufgrund fehlender wissenschaftlicher Evidenz sind derzeit Aussagen zur Infektiosität und Immunität nicht sicher möglich.

Stand 01.06.2021

Corona-Diagnostik Insights

HELIX HUB

Invalidenstraße 113
10115 Berlin
Tel.: +49 30 516 959-310
info@corona-diagnostik-insights.de

Impressum · Datenschutz

Corona-Diagnostik Insights ist ein Projekt der ALM Service GmbH.

© 2021 ALM Service GmbH
