# Supplementary figures and images for: A calibration of nucleic acid (PCR) by antibody (IgG) tests in Germany: the course of SARS-CoV-2 infections estimated
Source: Front Epidemiol. 2025 Oct 13;5:1592629. doi: 10.3389/fepid.2025.1592629 (PMC12554765; doi:10.3389/fepid.2025.1592629)

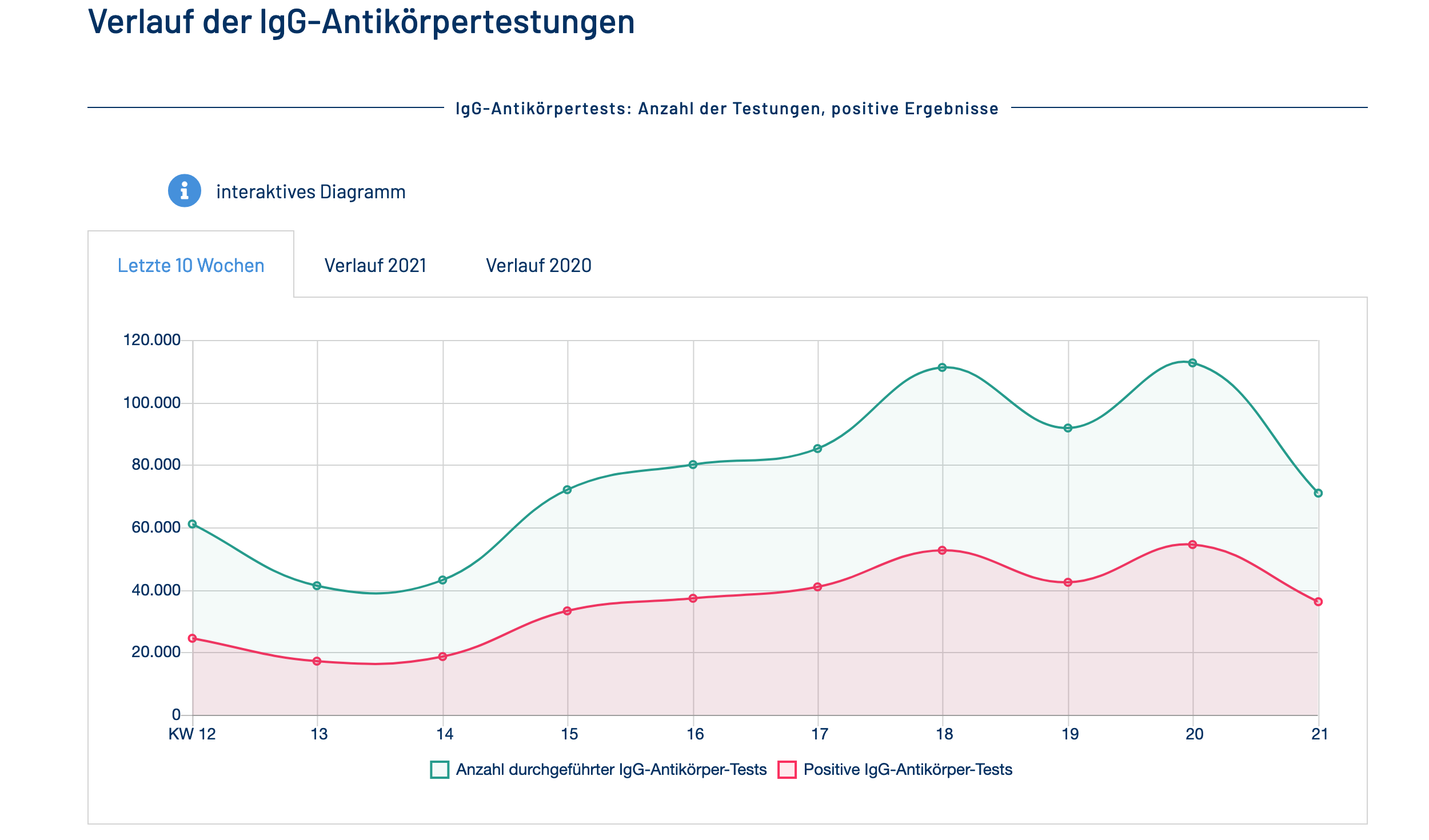

Supplement: Supplementary file 1 [file Datasheet1.zip › Supplement/ALM_GmbH_screen_shot_IgGAntikoerpertests_20211020.png]
